# Bilateral hemothorax secondary to adrenal hemorrhage

**DOI:** 10.11604/pamj.2024.47.130.42845

**Published:** 2024-03-22

**Authors:** Ashwin Karnan, Babaji Ghewade

**Affiliations:** 1 Department of Respiratory Medicine, Datta Meghe Institute of Higher Education and Research, Sawangi (Meghe), Wardha, Maharashtra, India

**Keywords:** Cortisol, hemorrhage, pleural effusion, adrenal malignancy

## Image in medicine

A 24-year-old male presented to our emergency department with complaints of difficulty in breathing, and left-sided abdominal and flank pain associated with generalized weakness for the past 6 days. Computed tomography showed bilateral pleural effusion of 51HU suggestive of hemothorax with an ill-defined heterogenous mass lesion in the left suprarenal region with free fluid in the abdomen. Ultrasound-guided (USG) pigtail was done for the left pleural effusion. Embolization of the bleeding vessel was done and the patient was taken for emergency laparotomy. Biopsy from the mass lesion showed adrenal cortical carcinoma. Adrenal hemorrhage is characterized by bleeding into the suprarenal glands. The adrenals derive rich arterial supply from three main arteries namely the superior, middle, and inferior suprarenal arteries. Biochemical markers include hyponatremia, hyperkalemia, hypoglycemia, anemia, and leukocytosis. Adrenal hemorrhage may be the initial presentation of an underlying adrenal mass lesion. Treatment modalities and prognosis are variable depending upon the etiology. Complications include retroperitoneal hemorrhage and hypovolemic shock.

**Figure 1:**
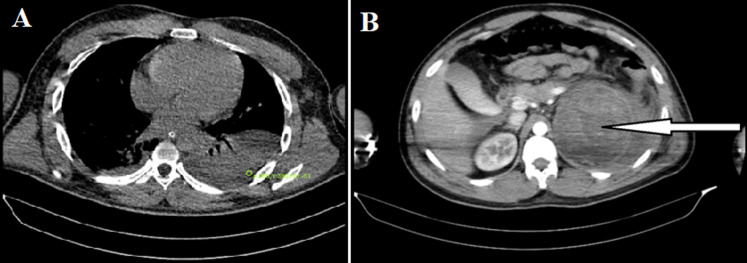
A) computed tomography showing bilateral pleural effusion with high attenuation 51HU; B) computed tomography showing heterogenous mass lesion in the left supra renal region marked by white arrow

